# Comparative effectiveness of a low-calorie diet combined with acupuncture, cognitive behavioral therapy, meal replacements, or exercise for obesity over different intervention periods: A systematic review and network meta-analysis

**DOI:** 10.3389/fendo.2022.772478

**Published:** 2022-08-26

**Authors:** Seo-Young Kim, In-Soo Shin, Young-Jae Park

**Affiliations:** ^1^ Department of Biofunctional Medicine & Diagnostics of Clinical Korean Medicine, Graduate School, Kyung Hee University, Seoul, South Korea; ^2^ Department of Transdisciplinary Security, Dongguk University, Seoul, South Korea; ^3^ Department of Biofunctional Medicine & Diagnostics, College of Korean Medicine, Kyung Hee University, Seoul, South Korea; ^4^ Department of Diagnosis and Biofunctional Medicine, College of Korean Medicine Kyung Hee University, Kyung Hee University Hospital at Gangdong, Seoul, South Korea

**Keywords:** low-calorie diet, acupuncture, cognitive behavior, meal replacement, exercise

## Abstract

**Objective:**

The aim of this study was to evaluate the comparative effectiveness of a low-calorie diet (LCD) combined with acupuncture, cognitive behavioral therapy (CBT), meal replacements (MR), and exercise on weight loss.

**Methods:**

The electronic databases MEDLINE, EMBASE, CENTRAL, CNKI, RISS, and KISS were searched systematically. Randomized controlled trials (RCTs) that directly compared the effect of a low-calorie diet (LCD)-combined acupuncture, CBT, and exercise and an MR-based diet on weight loss with LCD-alone for adults with simple obesity (body mass index [BMI] > 25) published before August 2021 were included in the study. Two investigators extracted and coded the data using a template. Any disagreements between investigators were resolved through discussion. Changes in BMI or weight were transformed to Hedges’ g values with a 95% CI, and network meta-analyses using a Bayesian random-effects model were conducted.

**Results:**

A total of thirty-two trials involving 3,364 patients were finally included in the study. The effect sizes of four interventions were medium, in the order of acupuncture (Hedges’ g = 0.48, 95% CI = 0.25 - 0.71), CBT (Hedges’ g = 0.42, 95% CI = 0.20 - 0.63), MR (Hedges’ g = 0.32, 95% CI = 0.19 - 0.45), and exercise (Hedges’ g = 0.27, 95% CI = 0.06 - 0.46).

In terms of intervention period, acupuncture was effective in the short period (≤ 12 weeks, Hedges’ g = 0.39, 95% CI = 0.12 - 0.67) and the long period (>12 weeks, Hedges’ g = 0.89, 95% CI = 0.37 - 1.40), whereas CBT (Hedges’ g = 0.51, 95% CI = 0.26 - 0.76) and exercise (Hedges’ g = 0.37, 95% CI = 0.12 - 0.59) were effective only in the long period. MR was effective only in the short period (Hedges’ g = 0.35, 95% CI = 0.18 - 0.53).

**Conclusions:**

This study suggests that acupuncture, CBT, MR, and exercise for simple obesity show a medium effect size, and their effectiveness differs according to the intervention period.

## Introduction

Overweight and obesity manifest as abnormal or excessive fat accumulation, which presents a health risk (1). An elevated body mass index (BMI) is a major risk factor for several chronic diseases such as type 2 diabetes, cardiovascular disease, osteoarthritis, and many cancers, including breast cancer in postmenopausal women; colon and rectal cancer; and adenocarcinoma of the esophagus, kidney, and pancreas (2–4). Reduction in total caloric intake may be a prerequisite for weight loss, and pharmacotherapy and bariatric surgery are used as adjuvant treatments to achieve this goal (5, 6). These techniques are used in combination with a calorie-restricted diet to achieve weight loss.

A previous meta-analysis reported that phentermine-topiramate and liraglutide had the highest chances of achieving at least 5% weight loss after 52 weeks (7). However, to date, there is little clinical evidence to show that obesity medication prevents cardiovascular disease or is safe in patients with obesity who are at a high risk of such diseases. Obesity medications, particularly sympathetic agents, should be carefully considered for such patients (8, 9). Bariatric surgery may be an option to improve morbid obesity-related conditions in patients who fail to respond to behavioral treatment (6, 10). However, mortality during or after bariatric surgery ranges from 0.1% to 1.1%, depending on the surgical method used (11), and the incidence of surgical complications such as hernia, intestinal stenosis, enterobrosia, and bleeding is 20% (12). Therefore, there is a need for non-pharmacological and non-surgical interventions, such as acupuncture, exercise, behavioral therapy, and meal replacement (MR), which are safe and effective for obesity (13–15).

Although some meta-analyses have reported the effectiveness of non-pharmacological and non-surgical interventions on obesity, the review did not strictly limit the control group and attributed diverse interventions to the control group, including diet, sham acupuncture, life modification (LM), or no treatment (16–21). For example, Zhang et al. reported that LM combined with acupuncture was more effective than LM alone (16). Kim et al. suggested that acupuncture combined with diet was more effective than sham acupuncture or LM (17). Curioni et al. reported that diet combined with exercise resulted in 20% greater initial weight loss than diet alone (18). Jacob et al. suggested that cognitive behavioral therapy (CBT)combined with a low-calorie diet (LCD) was more efficacious than CBT alone in increasing cognitive restraint, reducing emotional eating, and conferring weight loss (19). Astbury et al. and Heymsfield et al. reported that MR was more effective than diet alone for treating weight loss (20, 21).

When examining the effectiveness of interventions for obesity, researchers may encounter two drawbacks. First, if the intervention applied to the control group was not applied to the treatment group, it would be difficult to obtain a pure treatment effect. Furthermore, if the control groups differ among the different types of interventions, it is difficult to compare the comparative effectiveness of different interventions on obesity. If the control group is unified as an LCD group and the intervention group is defined as an LCD-combined intervention using network meta-analysis, the comparative effectiveness of each intervention on obesity may be evaluated (22). Together with differences in the effects of different interventions on obesity, previous studies have reported differences in the intervention period according to intervention type. This finding suggests that the intervention period may be associated with an intervention effect. Therefore, it is meaningful to investigate how the comparative effectiveness of each intervention is associated with the intervention period.

In summary, we aimed to analyze the comparative effects of acupuncture, CBT, MR, and exercise on simple obesity using network meta-analysis and to examine whether the intervention period affects the comparative effect of each intervention on simple obesity.

## Methods

### Data sources and search

**Figure 1:**
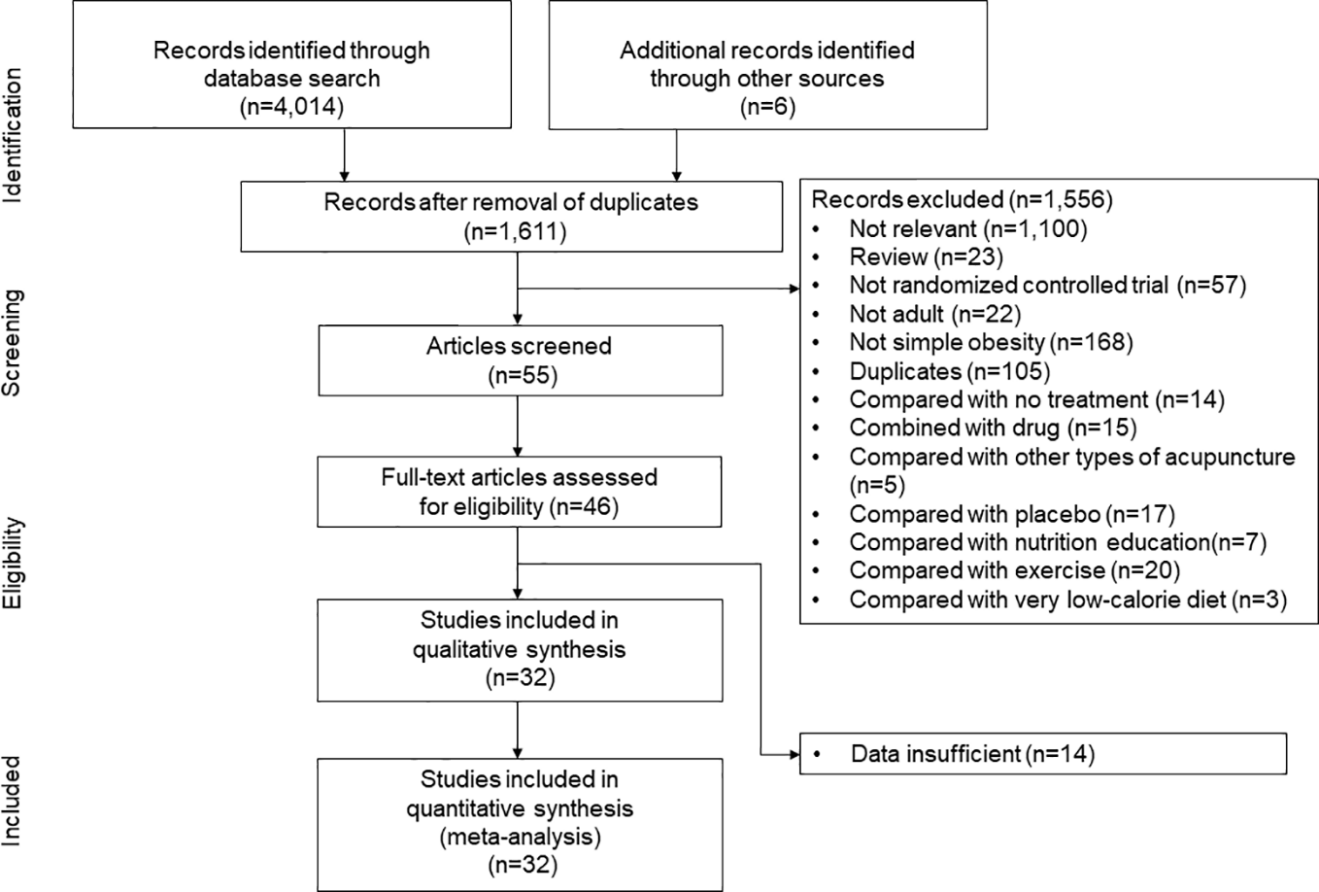
Study identification and selection. “Compared with other types of acupuncture” indicates the randomized controlled trials which compared the efficacy of low-calrorie diet (LCD)-combined catgut embedding and LCD-combined electroacupuncture, or the efficacy of LCD-combined acupuncture and LCD-combined catgut embedding.

We followed a standard systematic review protocol according to the PRISMA guidelines, which were recently adapted for network meta-analyses (**Figure 1**) (23). The following sources were searched for studies carried out in or before August 2021: MEDLINE, EMBASE, the Cochrane Central Register of Controlled Trials (CENTRAL), the China National Knowledge Infrastructure (CNKI), the Research Information Sharing Service, and the Korea Studies Information Service. Reviewers independently searched the articles using the following search terms: (“acupuncture” OR “acupressure OR “acupoint” OR “catgut” OR “embedding” OR “Cognitive Behavior* Therapy” OR “Cognitive Behavior* Treatment” OR “Cognitive Behavior* intervention” OR “meal replacement” OR “meal supplement” OR ((diet* OR “caloric restriction”) AND “exercise”)) AND (“weight loss” OR “simple obesity”). Any disagreements were resolved through discussion.

### Eligibility criteria and study selection

Randomized controlled trials (RCTs) that directly compared the effect of LCD alone with LCD-combined acupuncture, CBT, and exercise and an MR-based diet on weight loss in adults aged 18–65 years with simple obesity (BMI > 25) were included in the study. Further, RCTs that compared the effect of LCD-combined interventions such as LCD-combined CBT and LCD-combined exercise with other LCD-combined interventions were also included. However, RCTs in which a placebo, no treatment, and LCD combined with other interventions were assigned to the control group were excluded from the study. To synthesize the direct and indirect effects of the interventions, we conducted a lumping process in which treatment nodes were categorized to form single comparators. In our study, five interventions were combined: acupuncture plus LCD, CBT plus LCD, exercise plus LCD, MR-based LCD, and LCD alone. Among these interventions, acupuncture included laser acupuncture, manual acupuncture, and acupoint catgut embedding. CBT included psychoeducation, cognitive restructuring, and cognitive skills training on eating, weight, and emotions. Exercise training included any specific exercise prescription that included the mode of activity, frequency, intensity, and duration of the exercise. LCD was defined as a diet in which an energy deficit > 500 kcal/day was achieved with a dietary intake of 1,200 to 1,500 kcal/day in female participants and 1,500 to 1,800 kcal/day in male participants. An MR-based diet included calorie-restricted MR, in which a packaged food portion replaced one or more meals per day. However, MR-based diets combined with drugs were excluded from the study.

### Data extraction and quality assessment

A predefined data template was prepared after determination of the articles for inclusion. Two investigators (Kim SY and Shin YS) extracted and coded the data using a template. Included in the data template were the characteristics of the study (author name, publication year, journal, study design), participants demographic information (sample size, age, sex, body weight, and BMI), interventions (duration, frequency, types of therapy, and control group), and weight-related outcomes (mean changes and standard deviation in weight or BMI). Any disagreements between investigators were resolved through discussion. The methodological quality of the included studies was assessed using the Cochrane risk of bias tool (24), in which random sequence generation, allocation concealment, blinding of participants and personnel, blinding of outcome assessment, incomplete outcome data, selective reporting, and other biases were examined. In random sequence generation, using a random number table, computer random number generator, coin tossing, throwing dice, or shuffling cards was considered to satisfy low risk, whereas using odd or even date of birth, some rules based on date of admission, or clinical record number were considered as high risk. In allocation concealment, low risk was accepted when participants and investigators enrolling participants could not foresee assignment, because equivalent methods, including sequentially numbered or sealed envelopes or drug containers of identical appearance, were used to conceal allocation. A high risk of allocation concealment was determined when participants or investigators could foresee assignments because of an open random allocation schedule, date of birth, or case record number. In blinding of participants and personnel, a low risk was accepted when the outcome was not likely to be influenced by lack of blinding or not likely that the blinding was broken for participants and personnel, despite no blinding or incomplete blinding. A high risk of participant blinding was considered when the outcome was likely to be influenced by a lack of blinding. In outcome assessment blinding, low risk was accepted when the outcome was not likely to be influenced by lack of blinding or not likely that the blinding was broken for outcome assessors, whereas high risk was determined when the outcome was likely to be influenced by lack of blinding for outcome assessors. In incomplete outcome data, low risk was accepted when no missing outcome data or reported reasons for missing outcome data were unlikely to be related to the true outcome, such as survival data. A high risk was determined when dichotomous outcome data or reasons for missing outcome data likely to be related to true outcomes were reported. In selective reporting, low risk was accepted when the study protocol was available and prespecified (primary and secondary) outcomes were reported, whereas high risk was determined when prespecified outcomes were not reported or the study report failed to include a key outcome. Regarding other biases, low risk was accepted when the study appeared to be free of other sources of bias, whereas high risk was determined when the study had a potential source of bias related to the specific study design used. Among the seven risks of bias, unclear risk was determined when there was no reporting related to low or high risk for each assessment item.

### Statistical analyses

In our study, the primary outcome was a change in BMI (kg/m^2^) or weight (kg). Since all study outcomes were documented on a continuous scale, the concept of effect sizes was based on the standardized weighted mean differences with a 95% CI between the post-test and pre-test outcomes in the treatment and control groups. As the BMI scale differs from the weight scale, all study outcomes were transformed to Hedges’ g (24). Moreover, the effect sizes of small-sample studies tended to be overestimated, and Hedges’ g was used to adjust for sample size-related bias (25). Hedges’ g > 0.8 was considered “large,” that of 0.2–0.8 was considered “medium,” and Hedges’ g < 0.2 was considered “small” (25). Heterogeneity in the BMI or weight between studies was assessed using the Q-test and *I^2^* statistics. *I^2^* <40% was considered “non-important heterogeneity” and 30%-60% was considered “moderate heterogeneity” (24). Comprehensive meta-analysis software (version 3; Biostat Inc) was utilized to conduct a pairwise meta-analysis of the interventions in a direct, head-to-head manner using a random-effects model. The geMTC package (version 3.6.1) in R was used to conduct a network meta-analysis for the indirect effects of the interventions using an arm-based, Bayesian random-effects model.

Ranking of the intervention classes was presented using a league table, ranking probabilities, and rankograms (23). Network geometry was used to assess loop inconsistency assumptions according to closed intervention loops (26). The plausibility of the transitivity assumption was blindly evaluated by two investigators based on the design characteristics and methodologies of the included studies. Two investigators also evaluated clinical (patient, treatment) and methodological heterogeneity (study design and outcome measures). The consistency between the direct and indirect comparisons of all closed loops was evaluated using the ode-splitting method (23, 24).

## Results

### Study selection and characteristics

**Figure 2:**
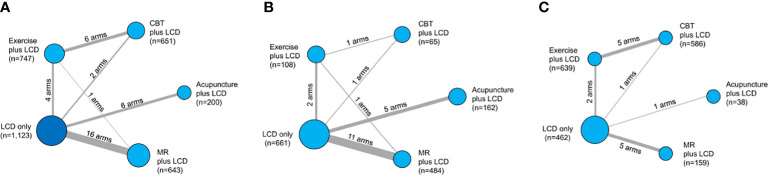
Network geometry of the five lumped interventions. Network geometry of the all study arms **(A)**, short-term (≤ 12 weeks) study arms **(B)**, and long- term (>12 weeks) study arms **(C)**. LCD, low-calorie diet; CBT, cognitive behavioral therapy; MR, meal replacement. The nodes correspond to the intervention type, and the edges correspond to the intervention arm between the two interventions. The size of the nodes indicates the number of patients, whereas the thickness of the edges indicates the number of intervention arms.

The initial search strategy identified 4,014 potential studies. Ultimately, 32 RCTs met the inclusion criteria and they were included in the final systematic review and meta-analysis (**Figure 1**). A total of 3,364 patients were included, with a mean age of 41.7 years. The proportion of females among the subjects was 78.1%, and the mean BMI was 33.0 kg/m^2^. The characteristics of the studies included in the network meta-analysis are summarized in **Table 1**. Thirty-five intervention arms were included, consisting of six types of direct pairwise comparisons: acupuncture plus LCD vs. LCD alone (27–30), CBT plus LCD vs. LCD alone (31, 35), exercise plus LCD vs. LCD alone (38–41), MR-based LCD vs. LCD alone (42–57), CBT plus LCD vs. exercise plus LCD (32–34, 36, 37), and MR-based LCD vs. exercise plus LCD (58). **Figures 2A-C** show the network geometry of all study arms, the short-term (≤ 12 weeks) study arms, and the long-term (>12 weeks) study arms. The network geometry formed two closed loops consisting of pairwise comparisons between interventions and a common comparator (LCD alone), as well as pairwise comparisons between interventions without a common comparator, and the possibility of loop inconsistency was excluded.

**Table 1:** Characteristics of the included randomized controlled trials.

Author (year)	Intervention class of treatment group	Intervention class of control group	Period(weeks)	Number of patients randomized	Number of patients analyzed	Age	Female (%)	Initial BMI	Outcome	Adverse events
Wozniak (2003) (27)	Acupuncture plus LCD	LCD alone	24	74	74	55.05	100	33.7	BMI	Undocumented
Jin (2009) (28)	Acupuncture plus LCD	LCD alone	8.5	80	80	30.5	80	28.44	BMI	None
Du (2011) (29)	Acupuncture plus LCD	LCD alone	12	64	64	50.05	100	30.61	BMI	Undocumented
Deng (2014a) (30)	Acupuncture plus LCD	LCD alone	10	60	60	32.5	88.33	NR	BW	Undocumented
Deng (2014b) (30)	Acupuncture plus LCD	LCD alone	10	60	60	32.5	91.67	NR	BW	Undocumented
Deng (2014c) (30)	Acupuncture plus LCD	LCD alone	10	60	60	32	91.67	NR	BW	Undocumented
Stahre (2007) (31)	CBT plus LCD	LCD alone	10	54	29	48.39	100	34.73	BW	Undocumented
Rodriguez (2009a) (32)	CBT plus LCD	Exercise plus LCD	24	47	45	45.4	100	36.39	BMI	Undocumented
Rodriguez (2009b) (32)	CBT plus LCD	Exercise plus LCD	24	58	57	45.4	100	35.79	BMI	Undocumented
Annesi (2012) (33)	CBT plus LCD	Exercise plus LCD	26	430	430	42.5	82.6	41.7	BW	Undocumented
Annesi (2013) (34)	CBT plus LCD	Exercise plus LCD	26	340	324	43.4	80.6	40.3	BMI	Undocumented
Jamal (2016) (35)	CBT plus LCD	LCD alone	24	194	151	40.05	72.7	32.4	BMI	Undocumented
Manzoni (2016) (36)	CBT plus LCD	Exercise plus LCD	6	106	102	35.63	100	42.24	BW	Undocumented
Palmeira (2017) (37)	CBT plus LCD	Exercise plus LCD	14	73	59	42.36	100	34.23	BMI	Undocumented
Skender (1996) (38)	Exercise plus LCD	LCD alone	48	84	36	NR	NR	NR	BW	Undocumented
McCrory (1999) (39)	Exercise plus LCD	LCD alone	1.5	44	44	31.7	47.72	25.35	BW	Undocumented
Nieman (2002) (40)	Exercise plus LCD	LCD alone	12	48	48	NR	NR	33.47	BMI	Undocumented
Philippou (2012) (41)	Exercise plus LCD	LCD alone	18	337	337	35	61.12	30.93	BW	Undocumented
Flechtner (2000) (42)	MR-based LCD	LCD alone	12	100	100	45.2	60	33.6	BW	Undocumented
Rothacker (2001) (43)	MR-based LCD	LCD alone	52	75	61	36.86	100	28.92	BW	None
Ahrens (2003) (44)	MR-based LCD	LCD alone	12	95	88	47.71	87.37	29.26	BW	None
Allison (2003) (45)	MR-based LCD	LCD alone	12	100	74	50.2	80	34.3	BW	Gas/indigestion (1.59 ± NR, p<0.05), taste: abnormal/metallic (0.3 ± NR, p<0.05), lethargy/no movement (0.19 ± NR, p<0.05)
Fisberg (2004) (46)	MR-based LCD	LCD alone	12	78	67	36.02	93.59	NR	BW	Transitory elimination of flatus (n=1)
Noakes (2004) (47)	MR-based LCD	LCD alone	24	66	42	48.14	NR	32.54	BW	None
Ashley (2007) (48)	MR-based LCD	LCD alone	52	96	70	38.25	100	29.3	BMI	Undocumented
Rohrer (2008) (49)	MR-based LCD	LCD alone	4	63	55	47.25	80	36.27	BW	None
Tsai (2009) (50)	MR-based LCD	LCD alone	12	120	120	43.15	85.83	32.35	BW	Diarrhea (0.59 ± 0.01, p<0.05), gas/indigestion (1.27 ± 0.16, p<0.05), sleep loss 32.7424(1.57 ± 0.73, p<121000.05)
Smith (2010) (51)	MR-based LCD	LCD alone	24	113	113	28.21	32.74	33.1	BMI	None120
Metzner (2011) (52)	MR-based LCD	LCD alone	12	105	87	49.7	100	31.2	BMI	Diarrhe24a(n=1)
Khoo (2013) (53)	MR-based LCD	LCD alone	12	48	46	40.5	0	32.66	BMI	Undocum6ented
König (2015) (54)	MR-based LCD	LCD alone	24	50	42	49	57.14	32.7	BMI	None12
Fuller (2016) (55)	MR-based LCD	LCD alone	6	76	76	42.25	53.95	30.35	BW	Gastroi8ntestinal distress and6 constipation or bloating (n=2)
Gulati (2017) (56)	MR-based LCD	LCD alone	12	122	122	37.54	57.38	30.35	BMI	Undocumented
Shih (2019) (57)	MR-based LCD	LCD alone	8	60	56	38.07	51.79	24.84	BMI	Undocumented
König (2014) (58)	MR-based LCD	Exercise plus LCD	6	42	42	54	61.9	32.8	BMI	Undocumented

BMI, body mass index; LCD, low-calorie diet; NR, not reported; BW, body weight; CBT, cognitive behavioral therapy; MR, meal replacement. The small letter attached to the published year indicates the number of trial arms within one randomized controlled trial.

### Pairwise meta-analysis findings

**Figure 3:**
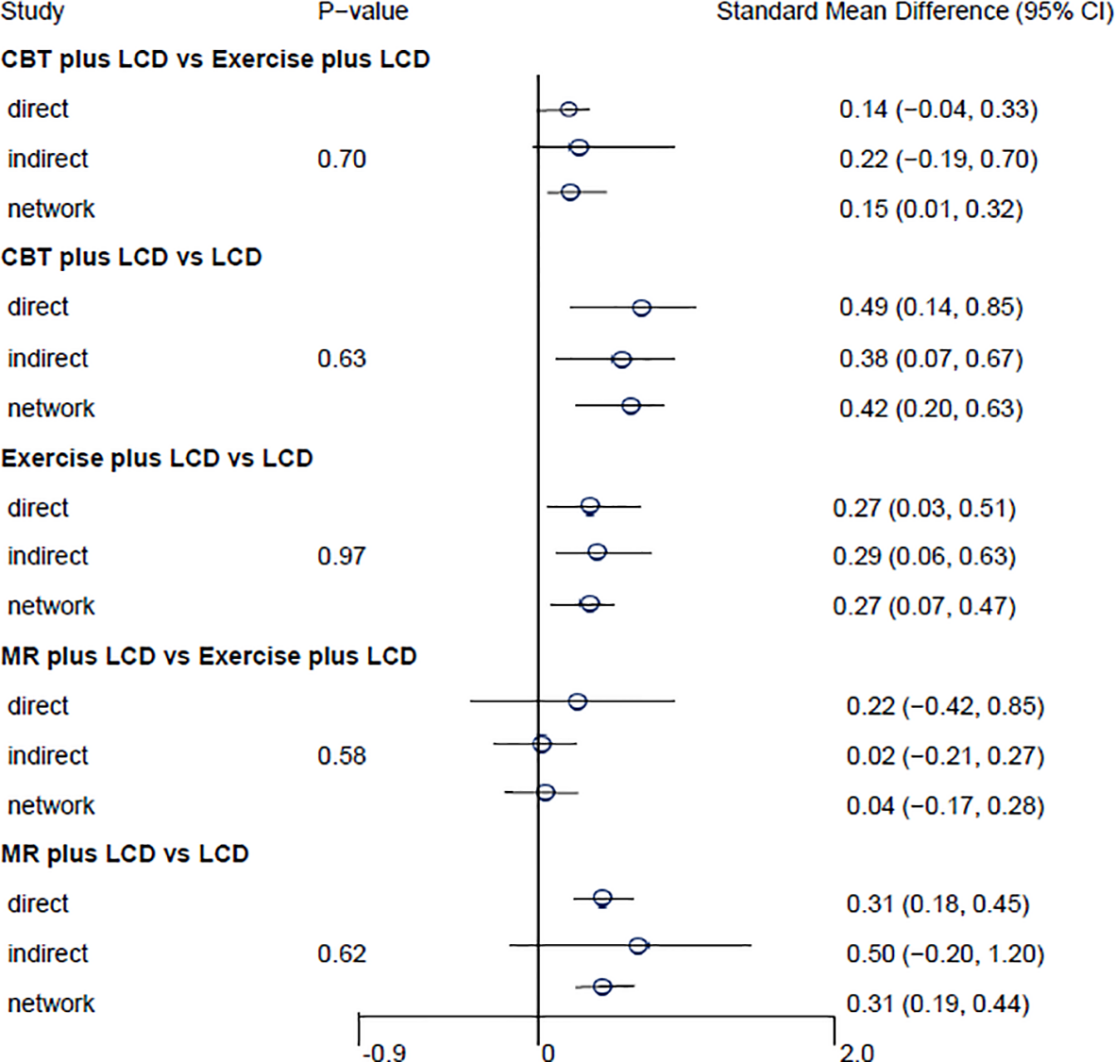
Forest plots of the five lumped interventions. LCD, low-calorie diet; CBT, cognitive behavioral therapy; MR, meal replacement.

The results of the direct pairwise meta-analysis are summarized in **Table 2**. Forest plots of the five lumped interventions are shown in **Figure 3**. All LCD-combined interventions were more effective than LCDs alone. The effect sizes on weight loss of LCD-combined acupuncture (Hedges’ g = 0.48, 95% CI = 0.24-0.71), LCD-combined CBT (Hedges’ g = 0.49, 95% CI = 0.14-0.85), LCD-combined exercise (Hedges’ g = 0.27, 95% CI = 0.03-0.51), and MR-based LCD (Hedges’ g = 0.31, 95% CI = 0.18-0.45) were higher than those of LCD alone. The direct effects on weight loss of LCD-combined CBT (Hedges’ g = 0.14, 95% CI = -0.04-0.45) and MR-based LCD (Hedges’ g = 0.22, 95% CI = -0.42-0.85) were not significantly different from those of LCD-combined exercise.

**Table 2:** Results of direct, indirect comparison and network meta-analysis.

Intervention		Comparison	Effect size and 95% confidence interval								
			Direct estimates			Indirect estimates			Network estimates		
			Point estimate	Lower limit	Upper limit	Point estimate	Lower limit	Upper limit	Point estimate	Lower limit	Upper limit
Acupuncture plus LCD	LCD alone		0.48	0.24	0.71	None	None	None	0.48	0.25	0.71
CBT plus LCD	LCD alone		0.49	0.14	0.85	0.38	0.07	0.67	0.42	0.20	0.63
Exercise plus LCD	LCD alone		0.27	0.03	0.51	0.29	-0.06	0.63	0.27	0.07	0.47
MR-based LCD	LCD alone		0.31	0.18	0.45	0.50	-0.20	1.20	0.31	0.19	0.44
CBT plus LCD	Exercise plus LCD		0.14	-0.04	0.33	0.22	-0.19	0.70	0.15	0.01	0.32
MR-based LCD	Exercise plus LCD		0.22	-0.42	0.85	-0.02	-0.21	0.27	0.04	-0.17	0.28
Acupuncture plus LCD	CBT plus LCD		None	None	None	0.06	-0.24	0.38	0.06	-0.24	0.38
Acupuncture plus LCD	Exercise plus LCD		None	None	None	0.20	-0.08	0.51	0.21	-0.08	0.51
Acupuncture plus LCD	MR-based LCD		None	None	None	0.17	-0.10	0.42	0.17	-0.10	0.42
CBT plus LCD	MR-based LCD		None	None	None	0.11	-0.14	0.34	0.11	-0.14	0.34

LCD, low-calorie diet; CBT, cognitive behavioral therapy; MR, meal replacement.

### Network meta-analysis findings

**Figure 4:**
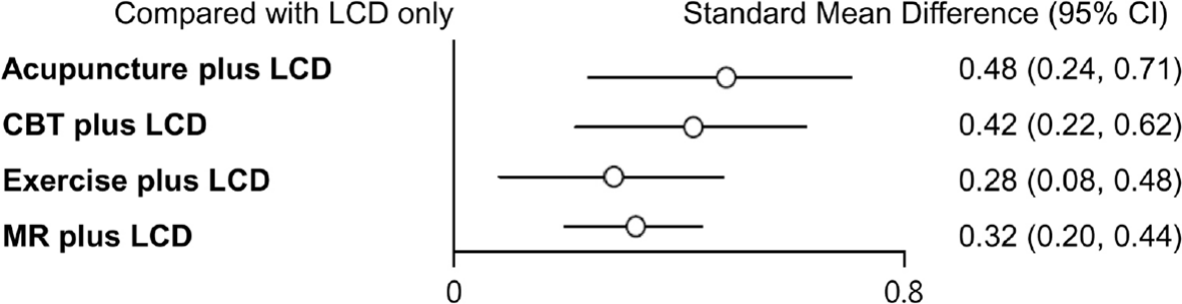
Forest plots of each intervention compared with LCD alone. LCD, low-calorie diet; CBT, cognitive behavioral therapy; MR, meal replacement.

Indirect comparisons are built on an assumption of transitivity, which refers to the similarity of one set of RCTs to another sets of RCTs in all important factors except the intervention comparison being made (26). Two investigators (Kim SY and Shin YS) independently assessed the transitivity of all RCTs, and the transitivity assumption was accepted. **Figure 4** shows the forest plots of each intervention compared with the LCD alone *via* a network meta-analysis. LCD combined acupuncture was found to be the most effective (Hedges’ g = 0.48, 95% CI = 0.25-0.71), followed by LCD-combined CBT (Hedges’ g = 0.42, 95% CI = 0.20-0.63), MR-based LCD (Hedges’ g = 0.32, 95% CI = 0.19-0.45), and LCD-combined exercise (Hedges’ g = 0.27, 95% CI =0.06-0.46). The league table showing the relative rankings of the interventions is listed in **Supplementary Table 1** in the supplement. The rankograms for each intervention are shown in **Supplementary Figure 1** in the supplement. The league table and rankograms indicated that LCD-combined acupuncture was the most effective for weight loss, followed by LCD-combined CBT, MR-based diet, and LCD-combined exercise. The numerical ranking probability is summarized in **Supplementary Table 2** in the supplement. LCD-combined acupuncture showed the highest probability of being ranked first in the first rank (63.70%), and LCD-combined CBT showed the highest probability of being ranked first in the second rank (48.98%). MR-based LCD (44.05%), LCD-combined exercise (61.22%), and LCD only (99.19%) showed the highest probability of being ranked first in the third, fourth, and fifth ranks, respectively.

### Heterogeneity test

The heterogeneity test results are presented in **Table 3**. There was no significant within-intervention heterogeneity. However, the Q (df = 34) value of the 35 study arms was 51.03 (p = 0.03), indicating significant inter-intervention heterogeneity. The *I^2^* value of all interventions was 33.37%, indicating a boundary between the low and moderate levels.

**Table 3:** Test of heterogeneity in the BMI or weight of pairwise meta-analysis assessed by Q-test and *I^2^* statistics.

Intervention type	Comparison	Numbersof studyarms	Hedges’ gvalue	Standard error	95% CI, lower limit	95% CI, upper limit	Q-value	df (Q)	P-value	*I^2^* (%)
Acupuncture plus LCD	LCD alone	6	0.48	0.10	0.29	0.68	6.88	5	0.23	27.34
CBT plus LCD	LCD alone	2	0.47	0.15	0.17	0.76	0.15	1	0.70	0.00
Exercise plus LCD	LCD alone	4	0.31	0.09	0.13	0.50	3.99	3	0.26	24.72
MR based LCD	LCD alone	16	0.30	0.06	0.19	0.42	23.28	15	0.08	35.57
CBT plus LCD	Exercise plus LCD	6	0.11	0.06	-0.01	0.24	3.89	5	0.57	0.00
MR based LCD	Exercise plus LCD	1	0.22	0.32	-0.41	0.85	0.00	0	1.00	0.00
Total within intervention							38.19	29	0.12	
Total between interventions							12.85	5	0.03	
Overall		35	0.28	0.04	0.21	0.34	51.03	34	0.03	33.37

LCD, low-calorie diet; CBT, cognitive behavioral therapy; MR, meal replacement; BMI, body mass index.

### Effectiveness on weight loss by intervention period

We examined whether the intervention period affected the effect size of each intervention, using intervention periods of 12 weeks or less (short-term) and over 12 weeks (long-term). **Figures 2B, C** show the network geometry in the short and long terms. **Table 4** summarizes the network meta-analysis results based on the intervention period. LCD-combined acupuncture was moderately effective in the short term (Hedges’ g = 0.39, 95% CI = 0.12-0.67), while LCD-combined acupuncture was highly effective in the long-term when compared with LCD alone (Hedges’ g = 0.89, 95% CI = 0.37-1.40). LCD-combined CBT in the long-term was moderately effective when compared with LCD alone (Hedges’ g = 0.51, 95% CI = 0.26-0.76), while LCD-combined CBT was not significantly effective in the short term (Hedges’ g = 0.23, 95% CI = -0.30-0.75). LCD-combined exercise was moderately effective in the long term (Hedges’ g = 0.37, 95% CI = 0.12- 0.59), while it was not significantly effective in the short-term (Hedges’ g = 0.11, 95% CI = -0.27-0.48). The MR-based diet was moderately effective in the short term (Hedges’ g = 0.35, 95% CI = 0.18-0.53), while it was not significantly effective in the long term (Hedges’ g = 0.22, 95% CI = -0.01-0.47). Non-standardized mean differences in BMI between LCD-combined interventions and LCD alone are summarized in **Supplementary Table 3** in the supplement.

**Table 4:** Network meta-analysis of the effect size on weight loss by intervention period.

Intervention	Comparison	Short-term period (≤ 12weeks)					Long-term period (> 12weeks)				
		Numbers of study arms	Hedges’ g value	Standard error	95% CIlower limit	95% CIupper limit	Numbers of study arms	Hedges’g value	Standard error	95% CIlower limit	95% CIupper limit
Acupuncture pus LCD	LCD alone	5 (28–30, 30)	0.39	0.14	0.12	0.67	1 (27)	0.89	0.26	0.37	1.40
CBT plus LCD	LCD alone	2 (31, 36)	0.23	0.27	-0.30	0.75	6 (32–35, 37)	0.51	0.13	0.26	0.76
MR based LCD	LCD alone	12 (42, 44–46, 49, 50, 52, 53, 55–58)	0.35	0.09	0.18	0.53	5 (43, 46, 48, 51, 56)	0.22	0.12	-0.01	0.47
Exercise plus LCD	LCD alone	4 (36, 39, 40, 58)	0.11	0.19	-0.27	0.48	7 (32–34, 37, 38, 41)	0.37	0.12	0.12	0.59

wks, weeks; LCD, low-calorie diet; SE, standard error; CBT, cognitive behavioral therapy; MR, meal replacement.

### Risk of bias and publication bias

**Figure 5:**
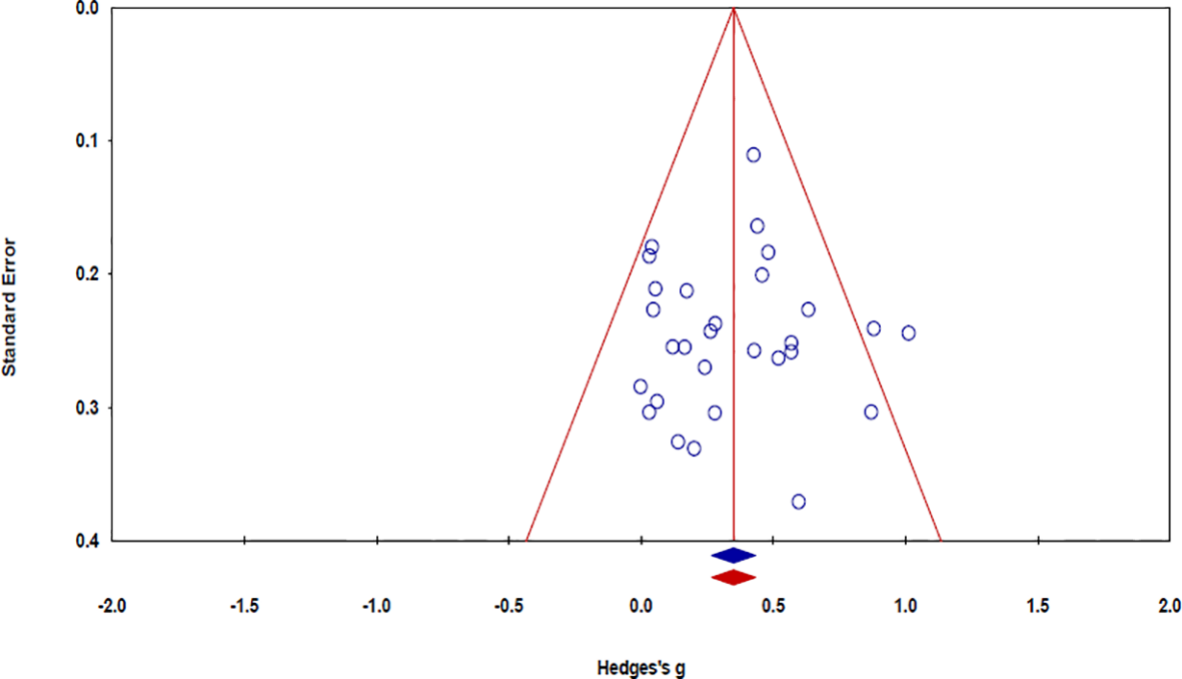
Funnel plot of the effect size.

The risk of bias in the 32 studies is summarized in **Table 5**. Twelve studies used adequate methods of sequence generation, such as a computerized random number generator (34, 37, 39, 45, 52, 55, 57), the blocked random method (30, 44, 56), website randomization (36), or coin flip (49). The remaining 20 studies failed to report a precise randomization method. Allocation concealment was conducted in seven studies using opaque sealed envelopes (53), computer-based allocations (34, 37, 39, 45, 55), or website randomization (36). The remaining 25 studies failed to specifically mention the concealment methods used therein. Regarding the patient blinding process, the risk of bias was high in six studies. The risk of bias was also high in five studies with regard to outcome assessor blinding. As the included studies were not placebo-controlled, it was difficult for them to be designed as double-blind trials. There was no evidence of a high risk of bias in the other studies. Publication bias was examined using a funnel plot with trim and fill. The funnel plot had a symmetrical distribution, indicating no publication bias (**Figure 5**) (59).

**Table 5:** Risk of bias of within individual studies.

Author (year)	Sequencegeneration	Allocationconcealment	Patientsblinded	Outcomeassessors blinded	Incompleteoutcome data	Selectivereporting	Otherbias	Summary
Wozniak (2003)	Unclear	Unclear	High	High	Low	Low	Low	High
Jin (2009)	Unclear	Unclear	High	High	Low	Low	Low	High
Du (2011)	Unclear	Unclear	High	High	Low	Low	Low	High
Deng (2014)	Low	Unclear	High	High	Low	Low	Low	High
Tanco (1998)	Unclear	Unclear	Unclear	Unclear	Low	Low	Low	Unclear
Ames (2005)	Unclear	Unclear	Unclear	Unclear	Low	Low	Low	Unclear
Stahre (2007)	Unclear	Unclear	Unclear	Low	Low	Low	Low	Unclear
Rodriguez (2009)	Unclear	Unclear	Unclear	Low	Low	Low	Low	Unclear
Annesi (2012)	Unclear	Unclear	Unclear	Low	Low	Low	Low	Unclear
Annesi (2013)	Low	Low	Unclear	Low	Low	Low	Low	Unclear
Jamal (2016)	Unclear	Low	High	High	Low	Low	Low	High
Manzoni (2016)	Low	Low	Unclear	Unclear	Low	Low	Low	Unclear
Palmeira (2017)	Low	Low	High	Low	Low	Low	Low	High
Skender (1996)	Unclear	Unclear	Low	Low	Low	Low	Low	Unclear
McCrory (1999)	Low	Low	Low	Low	Low	Low	Low	Low
Nieman (2002)	Unclear	Unclear	Low	Low	Unclear	Low	Low	Unclear
Philippou (2012)	Unclear	Unclear	Low	Low	Unclear	Unclear	Low	Unclear
Flechtner (2000)	Unclear	Unclear	Low	Low	Unclear	Low	Low	Unclear
Rothacker (2001)	Unclear	Unclear	Low	Low	Unclear	Low	Low	Unclear
Ahrens (2003)	Low	Unclear	Low	Low	Unclear	Low	Low	Unclear
Allison (2003)	Low	Low	Low	Low	Low	Low	Low	Low
Fisberg (2004)	Unclear	Unclear	Low	Low	Unclear	Low	Low	Unclear
Noakes (2004)	Unclear	Unclear	Low	Low	Low	Low	Low	Unclear
Ashley (2007)	Unclear	Unclear	Low	Low	Low	Low	Low	Unclear
Rohrer (2008)	Low	Unclear	Low	Low	Low	Low	Low	Unclear
Tsai (2009)	Unclear	Unclear	Low	Low	Unclear	Low	Low	Unclear
Smith (2010)	Unclear	Unclear	Low	Low	Low	Low	Low	Unclear
Metzner (2011)	Low	Unclear	Low	Low	Low	Low	Low	Unclear
Khoo (2013)	Unclear	Low	Low	Low	Low	Low	Low	Unclear
König (2015)	Unclear	Unclear	Low	Low	Low	Low	Low	Unclear
Fuller. (2016)	Low	Low	Low	Low	Low	Low	Low	Unclear
Gulati (2017)	Low	Unclear	Low	Low	Low	Low	Low	Unclear
Shih (2019)	Low	Unclear	Low	Low	Unclear	Low	Low	Unclear
König (2014)	Unclear	Unclear	Low	Low	Low	Low	Low	Unclear

### Adverse events

Of the 32 studies, seven reported that there were no adverse events (28, 43, 44, 47, 49, 51, 54), and five reported adverse events (45, 46, 50, 54, 55). The remaining 20 studies did not report any results regarding adverse events. The five studies that reported adverse events were related to an MR-based diet, and the adverse events reported were not serious. Allison et al. reported abdominal inflation, indigestion, abnormal taste, and lethargy (45). Fisberg et al. reported one case of a transitory elimination of flatus, which discontinued after 2 weeks of drug usage (46). Tsai et al. reported adverse effects of a green tea MR, including diarrhea, indigestion, and sleep loss (50). Metzner et al. reported that one patient was withdrawn from the study due to diarrhea (54). Fuller et al. reported two cases of gastrointestinal distress, constipation, and bloating (55).

## Discussion

To our knowledge, this study is the first to evaluate the comparative effectiveness of an LCD combined with acupuncture, CBT, MR, and exercise on weight loss. Our study revealed that LCD-combined acupuncture, CBT, exercise, and MR-based LCD were more effective in simple obesity than LCD alone. The effect sizes of LCD-combined acupuncture, CBT, MR-based LCD, and exercise were 0.48, 0.42, 0.32, and 0.27, respectively. Therefore, our study results suggest that these four LCD-combined interventions have an effect size of medium for weight loss. Another finding of this study was that the efficacy of these four interventions for obesity was associated with the intervention period. Therefore, the results of this study suggest that the intervention period should be considered together with the means of intervention when establishing a weight loss plan.

Acupuncture increases a number of molecules related to appetite, such as nesfatin-1 and cocaine- and amphetamine-regulated transcript peptides distributed in the appetite-associated hypothalamic nuclei and it decreases the secretion of digestive enzymes such as salivary amylase, serum pepsinogen, and gastric acid, thus inhibiting the function of gastrointestinal digestion and absorption (60). As a result, acupuncture suppresses appetite and reduces food intake, thus contributing to reduced emotional eating due to depression and anxiety. CBT has been reported to be efficacious for eating disorders by improving the conscious restriction of food intake and reducing depression and anxiety (19). CBT is beneficial for reducing general stress, distress due to medical conditions, chronic pain and fatigue, postnatal depression, and quality of life (61, 62). This complex mechanism of acupuncture and CBT may have resulted in a stronger effect on weight loss than LCD alone, when combined with LCD.

However, it was noticeable that not only effect sizes of acupuncture and CBT but also effect sizes of MR and exercise were all categorized into “medium” level (25). One possibility for this to occur is that efficacy of MR and exercise on simple obesity also increases significantly when combined with LCD. MR can be delivered within the community and purchased without a prescription (20), and exercise contributes to body weight and fat loss by increasing energy expenditure through physiological processes and cellular mechanisms that speed up glycogenolysis, glycolysis, fatty acid oxidation in muscle, and lipolysis in adipose tissue (63). Therefore, it appears that although the mechanisms of the four interventions were different, when combined with LCD, the therapeutic effect may have moderately increased compared to LCD alone. Nevertheless, the question remains as to why the synergistic effect of the four interventions did not show a “large” effect size when combined with LCD. One possibility for this is that since LCD itself has an effect on simple obesity, even if acupuncture, CBT, MR, or exercise intervention is combined with LCD, the synergistic effect will be limited to “medium” level (64).

Our study also revealed that the efficacy of the four LCD-combined interventions for simple obesity differed depending on the intervention period. LCD-combined acupuncture was effective both in the short- and long-term intervention periods; however, the effect size of intervention in the long-term period was approximately two-fold that in the short-term. LCD-combined CBT and exercise were effective in the long-term; however, they were not significantly effective in the short term. MR-based LCD was effective only in the short term, while it was not significantly effective in the long term. Therefore, considering the association between the effect size of each intervention and intervention period, a differentiated plan for each intervention for simple obesity may be established. For example, LCD-combined acupuncture is effective for simple obesity in the short term; however, its efficacy may increase in the long-term. Therefore, acupuncture may be recommended primarily for patients with simple obesity, in whom the duration of treatment cannot be definitively determined. In contrast, LCD-combined CBT and exercise are effective for simple obesity only in the long term. Therefore, with regard to these two interventions, patients with simple obesity should be given sufficient information about the intervention period. To understand the reason why MR-based LCD was not effective in long-term treatment, one possibility could be that the compliance of patients to an MR-based LCD may have been reduced for the long-term. Therefore, it would be undesirable to exceed 12 weeks for MR-based LCD. Some adverse events, including abdominal inflation, indigestion, abnormal taste, and sleep loss, have been reported only for an MR-based LCD. Therefore, together with the intervention period, gastrointestinal and neurological adverse events should be monitored or relieved when applying MR-based LCD to patients with simple obesity.

The heterogeneity test revealed significant differences in total between-intervention heterogeneity. In particular, the *I^2^* levels of LCD-combined acupuncture, exercise, and MR-based LCD were higher than those of LCD-combined CBT with LCD alone or LCD-combined exercise, and differences in *I^2^* levels between the higher and lower heterogeneity groups may have resulted in significant heterogeneity of the overall interventions. Large sample sizes have been reported to lower the *I^2^* level (65). However, there were 6 and 4 study arms for LCD-combined acupuncture and exercise versus 16 for MR-based LCD. Therefore, the number of study arms mainly contributed to the increase in overall intervention heterogeneities in this study. One possibility is that the study process itself differed within the acupuncture, exercise, or MR trials and between small or large numbers of study arms.

This systematic review and network meta-analysis have several limitations. The methodological quality of the included RCTs was low on account of insufficient number of patients and outcome assessors for double-blinding. The comparative efficacy of the four diet-combined interventions was examined only for people with simple obesity, and it is hence necessary to examine whether this result may be consistent with those of patients with severe obesity or secondary obesity. This study did not consider participants with no treatment as a control group, and it is challenging to examine the pure effect of acupuncture, CBT, MR, and exercise on simple obesity with no treatment as a control group. This study did not include other non-pharmacological and non-surgical interventions, such as massage, cupping, and bloodletting interventions for obesity. Therefore, further studies are needed to examine the comparative effectiveness of LCD combined with other intervention types than acupuncture, CBT, MR, and exercise for simple obesity.

In conclusion, this network meta-analyses, including 3,364 patients with simple obesity, suggest that acupuncture, CBT, MR, and exercise combined with LCD had the effect sizes of medium level (Hedges’ g ranging from 0.21 to 0.48) for weight loss, compared with LCD alone. Moreover, the efficacy of the four LCD-combined interventions was associated with the intervention period. Further studies are needed to overcome the limitations of simple obesity, control groups, and other interventions such as massage, cupping, and bloodletting interventions.

## Data availability statement

The original contributions presented in the study are included in the article/**Supplementary Material**. Further inquiries can be directed to the corresponding author.

## Author contributions

Study concept and design: S-YK and Y-JP. Acquisition of data: S-YK. Analysis and interpretation of data: S-YK and Y-JP. Drafting of the manuscript: S-YK. Critical revision of the manuscript for important intellectual content: Y-JP. Statistical analysis: S-YK and I-SS. Study supervision: Y-JP. All authors contributed to the article and approved the submitted version.

## Conflict of interest

The authors declare that the research was conducted in the absence of any commercial or financial relationships that could be construed as a potential conflict of interest.

## Publisher’s note

All claims expressed in this article are solely those of the authors and do not necessarily represent those of their affiliated organizations, or those of the publisher, the editors and the reviewers. Any product that may be evaluated in this article, or claim that may be made by its manufacturer, is not guaranteed or endorsed by the publisher.

## Supplementary material

Supplementary Figure 1 Rankograms of each intervention. LCD, low-calorie diet; CBT, cognitive behavioral therapy; MR, meal replacement.
