# Supplementary figures and images for: Comparative effectiveness of a low-calorie diet combined with acupuncture, cognitive behavioral therapy, meal replacements, or exercise for obesity over different intervention periods: A systematic review and network meta-analysis
Source: Front Endocrinol (Lausanne). 2022 Aug 26;13:772478. doi: 10.3389/fendo.2022.772478 (PMC9458910; doi:10.3389/fendo.2022.772478)

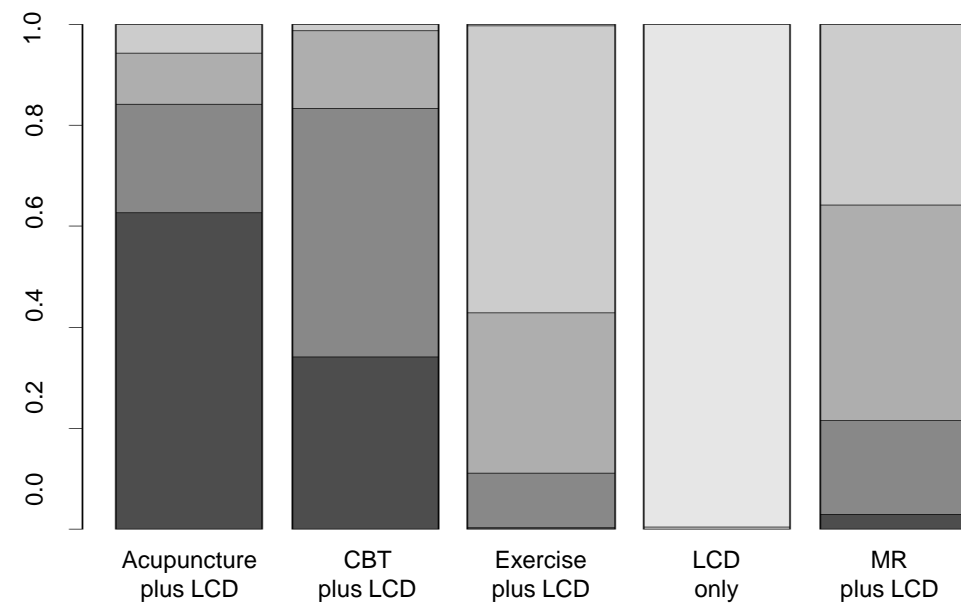

Supplement: Supplementary Figure 1 — Rankograms of each intervention. LCD, low-calorie diet; CBT, cognitive behavioral therapy; MR, meal replacement. [file DataSheet_1.pdf]
