# Supplementary material for: Comparative effectiveness of a low-calorie diet combined with acupuncture, cognitive behavioral therapy, meal replacements, or exercise for obesity over different intervention periods: A systematic review and network meta-analysis
Source: Front Endocrinol (Lausanne). 2022 Aug 26;13:772478. doi: 10.3389/fendo.2022.772478 (PMC9458910; doi:10.3389/fendo.2022.772478)
Supplement: Supplementary file 3 [file Table_1.docx]

**Supplementary Table 1.** League table of the included trials

| Acupuncture plus LCD |  |  |  |  |
| --- | --- | --- | --- | --- |
| 0.06 (-0.24, 0.38) | CBT plus LCD |  |  |  |
| 0.17 (-0.10, 0.42) | 0.11(-0.14, 0.34) | MR-based LCD |  |  |
| 0.21 (-0.08, 0.51) | 0.15 (-0.01, 0.32) | 0.04 (-0.17, 0.28) | Exercise plus LCD |  |
| 0.48 (0.25, 0.71) | 0.42 (0.20, 0.63) | 0.32 (0.19, 0.45) | 0.27 (0.06, 0.46) | LCD alone |

LCD, low-calorie diet; CBT, cognitive behavioral therapy; MR, meal replacement.
