# Supplementary material for: Comparative effectiveness of a low-calorie diet combined with acupuncture, cognitive behavioral therapy, meal replacements, or exercise for obesity over different intervention periods: A systematic review and network meta-analysis
Source: Front Endocrinol (Lausanne). 2022 Aug 26;13:772478. doi: 10.3389/fendo.2022.772478 (PMC9458910; doi:10.3389/fendo.2022.772478)
Supplement: Supplementary file 4 [file Table_2.docx]

**Supplementary Table 2.** Ranking probabilities

| Intervention type | Rank | | | | |
| --- | --- | --- | --- | --- | --- |
|  | 1 | 2 | 3 | 4 | 5 |
| Acupuncture plus LCD | 0.6370 | 0.2437 | 0.0790 | 0.0404 | <0.0001 |
| CBT plus LCD | 0.3233 | 0.4900 | 0.1693 | 0.0173 | 0.0004 |
| Exercise plus LCD | 0.0036 | 0.0657 | 0.3109 | 0.6122 | 0.0077 |
| LCD only | <0.0001 | <0.0001 | 0.0005 | 0.0077 | 0.9919 |
| MR-based LCD | 0.0362 | 0.2009 | 0.4405 | 0.3225 | <0.0001 |

LCD, low-calorie diet; CBT, cognitive behavioral therapy; MR, meal replacement. The shaded cells indicate the rank with the highest probability for each intervention.
