# Supplementary material for: Comparative effectiveness of a low-calorie diet combined with acupuncture, cognitive behavioral therapy, meal replacements, or exercise for obesity over different intervention periods: A systematic review and network meta-analysis
Source: Front Endocrinol (Lausanne). 2022 Aug 26;13:772478. doi: 10.3389/fendo.2022.772478 (PMC9458910; doi:10.3389/fendo.2022.772478)
Supplement: Supplementary file 5 [file Table_3.docx]

**Supplementary Table 3**. Non-standardized mean differences in BMI between LCD-combined interventions and LCD alone

| Intervention type | Numbers of  study arms | BMI (kg/m^2^) | Standard  error | 95% CI,  lower limit | 95% CI,  upper limit |
| --- | --- | --- | --- | --- | --- |
| LCD-combined acupuncture | 3^27,28,29^ | 1.86 | 0.39 | 1.10 | 2.60 |
| LCD-combined CBT | 5^32a,23b,34,35,37^ | 0.74 | 0.27 | 0.21 | 1.30 |
| MR-based LCD | 8^48,51~54,56~58^ | 0.53 | 0.16 | 0.21 | 0.86 |
| LCD-combined exercise | 6^32a,32b,34,37,40,58^ | -0.06 | 0.33 | -0.05 | 0.67 |

LCD, low-calorie diet; CBT, cognitive behavioral therapy; MR, meal replacement; CI, confidence interval; BMI, body mass index. The negative mean differences of BMI in LCD-combined exercise are because changes in BMI were reported mostly in the short-term studies where the effectiveness of LCD-combined exercise was not significant. Wks, weeks; LCD, low-calorie diet; CBT, cognitive behavioral therapy; MR, meal replacement.
